# Initial outcomes of couple HOPES: A guided online couple intervention for PTSD and relationship enhancement

**DOI:** 10.1016/j.invent.2021.100423

**Published:** 2021-06-24

**Authors:** Skye Fitzpatrick, Anne C. Wagner, Alexander O. Crenshaw, Sonya Varma, Kristen M. Whitfield, Robert Valela, Alyssa A. Di Bartolomeo, Lindsay Fulham, Cait Martin-Newnham, Desiree H. Mensah, Alexis Collins, Meredith S.H. Landy, Leslie Morland, Brian D. Doss, Candice M. Monson

**Affiliations:** a Department of Psychology, York University, 4700 Keele St., Toronto, ON M3J 1P3, Canada; b Department of Psychology, Ryerson University, 350 Victoria St., Toronto, ON M5B 2K3, Canada; c Remedy, 703 Bloor St. W, #201, Toronto, ON M6G 1L5, Canada; d MindBeacon, 175 Bloor St. E., Toronto, ON M4W 358, Canada; e VA San Diego Healthcare System, San Diego, CA, USA and University of California San Diego, La Jolla, 3350 La Jolla Village Dr., San Diego, CA 92161, United States of America; f University of Miami, 1320 S Dixie Hwy, Coral Gables, FL, 33146, United States of America

**Keywords:** Posttraumatic stress disorder, Couple, Online, Intervention, Satisfaction

## Abstract

Couple HOPES (Helping Overcome PTSD and Enhance Satisfaction) is a guided, online couple intervention adapted from Cognitive-Behavioral Conjoint Therapy for posttraumatic stress disorder (PTSD). It was created to overcome a range of barriers to accessing evidence-based treatments for PTSD and the intimate relationship problems associated with it. This manuscript describes initial outcomes of the intervention in a series of 10 couples. Participants were military, veteran and first responders with probable PTSD and their intimate partners. Couples completed the program and measurements of PTSD, relationship satisfaction, and secondary outcomes at pre-, mid-, and post-intervention. Mean satisfaction for the program was high and it was completed by seven of ten couples. Participants with PTSD evidenced significant and large pre- to post-intervention effect size improvements in PTSD symptoms (*g* = 0.80) and perceived health (*g* = 1.13). They also exhibited non-significant but medium effect size pre- to post-intervention improvements in quality of life (*g* = 0.62), and depression (*g* = 0.53), and small effect size pre- to post-intervention improvements in argumentativeness (*g* = 0.43), anger (*g* = 0.31), and anxiety (*g* = 0.31). Partners reported significant and moderate pre- to post-intervention effect size improvements in relationship satisfaction *(g* = 0.68*),* and medium but not significant effect size improvements in accommodation of PTSD (*g* = 0.56). Results provide initial support for the feasibility, acceptability, and efficacy of Couple HOPES for improving PTSD and relationship satisfaction. However, more testing in larger samples, including with randomized controlled designs, is needed.

## Highlights

• Couple HOPES is an online, self-help couple intervention for PTSD.
• Feasibility, acceptability, and efficacy of Couple HOPES was tested with 10 couples.
• 10 couples with a military member/veteran/first responder with PTSD participated.
• Couple HOPES was feasible, acceptable, and resulted in reductions in PTSD symptoms.
• It improved relationship satisfaction for partners and several secondary outcomes.

## 1 Introduction

Posttraumatic stress disorder (PTSD) is prevalent, debilitating, and associated with relationship (Taft et al., 2011) and intimate partner (Lambert et al., 2012) distress. Military members, veterans, and first responders (MMVFR) have particularly elevated PTSD symptoms and rates (e.g., Creamer et al., 2011; Kleim and Westphal, 2011; Thompson et al., 2016) and higher occurrences of comorbid mental health problems – especially depression (Stander et al., 2014), anxiety (Maguen et al., 2012), alcohol/substance use problems (Norman et al., 2018; Seal et al., 2011), psychosocial impairment (Rona et al., 2009), anger (Gonzalez et al., 2016), and guilt (Owens et al., 2009). Importantly, MMVFR also experience particularly strong associations between PTSD and intimate relationship problems (Taft et al., 2011; McFarlane and Bookless, 2001). Indeed, intimate relationship dysfunction is also a risk factor for poor outcomes in individual PTSD treatment (e.g., Monson et al., 2005; Tarrier et al., 1999). Consequently, dyadic PTSD interventions such as Cognitive-Behavioral Conjoint Therapy (CBCT) for PTSD aim to simultaneously improve PTSD and relationship functioning (Monson and Fredman, 2012).

CBCT for PTSD is 15-session psychotherapy delivered conjointly to people with PTSD and their significant others that is designed to simultaneously treat PTSD and enhance relationship functioning and partner mental health. CBCT progresses through three phases: 1) Psychoeducation regarding the nature of PTSD and safety building in relationships; 2) Enhancing relationship functioning through communication skills training and undermining PTSD-related avoidance; and 3) Dyadic cognitive interventions targeting PTSD-related beliefs (Monson and Fredman, 2012). Several uncontrolled and controlled trials support the efficacy of CBCT for PTSD in improving patient- and partner-rated PTSD symptoms, relationship satisfaction, and broader psychosocial outcomes (see Liebman et al., 2020 for review). However, several barriers limit the uptake of face-to-face psychotherapies such as CBCT for PTSD, including availability and stigma regarding mental health treatment, as well as time, geographical, and financial constraints (Kazdin and Blase, 2011; Schnyder et al., 2017). Such barriers may be compounded by the COVID-19 pandemic, wherein social distancing prohibits in-person access of services (Johnson et al., 2021). This study describes the initial feasibility, acceptability, and efficacy of Couple HOPES (Helping Overcome PTSD and Enhance Satisfaction) – a guided online couple intervention adapted from CBCT for PTSD that was designed to overcome its access barriers.

Digital interventions offer promise as an effective intervention model because they have the potential to deliver low-cost, scalable, and accessible evidence-based interventions unhindered by aforementioned access barriers (Kazdin, 2008; Kuester et al., 2016). Several internet-delivered PTSD interventions have been developed and shown to be efficacious, especially ones that draw on cognitive behavioral therapies (CBT; e.g., Brief et al., 2013; Engel et al., 2015; Hirai and Clum, 2005; Ivarsson et al., 2014; Kersting et al., 2011, Kersting et al., 2013; Knaevelsrud and Maercker, 2007; Knaevelsrud et al., 2015; Kuhn et al., 2017; Krupnick et al., 2017; Lange et al., 2001, Lange et al., 2003; Lewis et al., 2017; Littleton et al., 2016; Litz et al., 2007; Miner et al., 2016; Spence et al., 2011, Spence et al., 2014; Steinmetz et al., 2012; Wagner et al., 2006; Wang et al., 2013) and expressive writing therapies (e.g., Beyer, 2011; Hirai et al., 2012; Possemato et al., 2010; Stockton et al., 2014). Meta-analyses suggest that online PTSD interventions exhibit a moderate effect size improvement in PTSD outcomes compared to passive control conditions (Kuester et al., 2016; Sijbrandij et al., 2016). Although some meta-analyses suggest that online CBTs exhibit a small effect size advantage compared to active controls (Sijbrandij et al., 2016), others suggest that specific active online interventions do not differ from each other (Kuester et al., 2016). However, various components of online PTSD interventions influence its efficacy. One meta-analysis suggested that inclusion of some type of support alongside the intervention (e.g., therapeutic support, coaching) significantly improves PTSD outcomes compared to interventions without such support, with a large effect size difference (Sijbrandij et al., 2016). Further, although another meta-analysis did not find significant effects between these two types of interventions (Kuester et al., 2016), online PTSD interventions without support showed a moderate effect size improvement in PTSD outcomes over passive control conditions, whereas interventions with support showed a large effect size difference in the same comparison (Kuester et al., 2016). These findings generally suggest that augmenting online PTSD interventions with support may optimize outcomes. Further, interventions with more modules may yield stronger outcomes compared to control conditions than those with less modules, although the significance of comparisons between these two intervention types are also mixed across meta-analyses (Kuester et al., 2016; Sijbrandij et al., 2016). Other intervention components (e.g., inclusion of multimedia versus not, use of exposure versus not) and sample characteristics (e.g., community versus clinical samples, PTSD diagnosis versus elevated PTSD symptoms) have not been shown to moderate outcomes (e.g., Kuester et al., 2016; Sijbrandij et al., 2016). Although low-cost, internet-based PTSD interventions are efficacious, they focus on treating individuals rather than the relational context in which PTSD is often embedded, ignoring intervention targets that are both worthy of clinical attention in and of themselves and contribute to PTSD maintenance (i.e., relationship functioning; Wagner et al., 2016).

To address this gap and building on research on online PTSD interventions, we developed Couple HOPES (Helping Overcome PTSD and Enhance Satisfaction) - an online, guided couple intervention delivered to individuals with PTSD and their intimate partners to reduce PTSD symptoms and enhance relationship satisfaction (www.couplehopes.com). This program incorporates coaches who facilitate engagement and troubleshoot couples' adherence to the program through secure messaging and brief videoconferencing calls (Monson et al., in press). Such coaching was included in light of aforementioned research showing that online PTSD interventions with support show superior outcomes to those that do not. Given that MMVFR show particularly elevated rates of PTSD (e.g., Creamer et al., 2011; Kleim and Westphal, 2011; Thompson et al., 2016) and relationship problems (Taft et al., 2011; McFarlane and Bookless, 2001), the present study tested the preliminary feasibility, acceptability, and efficacy of Couple HOPES in a case series consisting of MMVFR with PTSD and their intimate partners. Primary outcomes of interest were the reduction of PTSD symptoms and the enhancement of relationship satisfaction.

Given the widespread effects and correlates of PTSD in MMVFR, we also sought to examine whether Couple HOPES improves PTSD-relevant secondary outcomes across three domains: common comorbid problems (depression, anxiety, alcohol/substance use), interpersonal functioning/psychosocial impairment, and PTSD-related negative emotions (trauma-related guilt, anger). As well, given that the accommodation of partner's PTSD symptoms (e.g., supporting or “working around” trauma-related avoidance) is an established maintenance factor for PTSD (Fredman et al., 2014), we examined whether Couple HOPES improved partner-related accommodation as a secondary outcome.

We hypothesized that Couple HOPES would be highly feasible (i.e., high program completion rates), acceptable (i.e., high ratings of program satisfaction), and would result in improvements in self- and collateral-report ratings of PTSD, both couple member's relationship satisfaction, and related secondary outcomes.

## 2 Material and methods

### 2.1 Participants

The pilot case series sample consisted of 10 adult intimate dyads wherein one partner was a MMVFR with clinically significant self-reported PTSD symptoms who were recruited during the COVID-19 pandemic. Table 1 includes a description of the participants in the study. Specific inclusion criteria for the study involved one member of the couple: (1) being a Canadian MMVFR; (2) who experienced a Diagnostic and Statistical Manual of Mental Disorders, 5th edition (DSM-5; APA, 2013) Criterion A traumatic event (APA, 2013); and (3) has PTSD symptoms consistent with a probable PTSD diagnosis (i.e., a total score ≥33 on the Posttraumatic Checklist-5; Bovin et al., 2016; Weathers et al., 2013; Wortmann et al., 2016). Traumatic events did not need to occur in the context of the MMVFR's occupation for participants to be deemed eligible for study participation. Exclusion criteria involved either member of the couple: (1) endorsing elevated suicide risk; (2) not being willing to complete intervention modules together; (3) not having access to high-speed internet; (4) not being willing to have coaching sessions audio- or video-recorded; or (5) reporting the occurrence of severe intimate partner violence in the past year. Couples were also excluded if both members met the PTSD symptom inclusion criteria (i.e., dual probable PTSD). Participants were allowed to begin or continue outside mental health treatment during this study.

**Table 1:** Demographic data for all participants.

		Participants with PTSD		Intimate partners	
		ITT sample	Completer sample	ITT sample	Completer sample
Age		47.30 (10.86)	43.00 (7.51)	46.40 (10.95)	41.14 (7.11)
Gender	Male	70%	85.71%	30%	14.29%
	Female	20%	14.29%	70%	85.71%
	Non-Binary	10%	0%	0%	0%
Ethnicity	White/Caucasian/European Origin	90%	100%	90%	100%
	Other Asian or other Asian Canadian	10%	0%	0%	0%
	Bi-racial/Multi-racial	0%	0%	10%	0%
Highest Level of Education	Some High School	10%	14.29%	0%	0%
	High School Graduate	0%	0%	40%	28.57%
	Some College/university	10%	14.29%	10%	14.29%
	College Diploma	40%	42.86%	30%	28.57%
	Undergraduate Degree	30%	14.29%	10%	14.29%
	Masters Degree	10%	14.29%	10%	14.29%
Individual Annual Income	$15,000–$24,999	0%	0%	22.22%	33.33%
	$25,000–$34,999	20%	0%	11.11%	16.67%
	$35,000–$49,999	0%	0%	22.22%	16.67%
	$50,000–$74,999	30%	42.86%	22.22%	16.67%
	$75,000–$99,999	30%	28.57%	22.22%	16.67%
	$100,000–$249,999	20%	28.57%	0%	0%
Household Annual Income	$15,000–$24,999	0%	0%	0%	0%
	$25,000–$34,999	0%	0%	0%	0%
	$35,000–$49,999	0%	0%	0%	0%
	$50,000–$74,999	10%	14.29%	11.11%	16.67%
	$75,000–$99,999	30%	28.57%	33.33%	33.33%
	$100,000–$249,999	60%	57.14%	55.56%	50%
Current MMVFR Status	Veteran/Former First Responder	60%	71.43%	10%	0%
	Royal Canadian Mounted Police	20%	28.57%	0%	0%
	Military Service Member	20%	14.28%	10%	14.29%
	First Responder and Related Professions	30%	14.28%	0%	0%
	Not Applicable	0%	0%	80%	85.71%
Relationship Status	Married	90%	85.71%	90%	85.71%
	Common Law (Cohabitation one year or more)	10%	14.29%	10%	14.29%
Average Relationship Length in Years (SD)		14.98 (8.37)	15.96 (9.86)	15.20 (8.34)	15.83 (10.72)
Baseline Current Treatment	Individual therapy or counseling, with or without medications	80%	100%	10%	0%
	Family or Couples Therapy	10%	14.29%	0%	0%
	Self-help (e.g., Alcoholics Anonymous)	0%	0%	10%	14.29%
	Group Therapy	10%	0%	0%	0%
	Medications Only	10%	0%	10%	14.29%
	Not Applicable	10%	0%	70%	71.43%

*Notes.* ITT = Intent-to-treat; PTSD = Posttraumatic Stress Disorder; MMVFR = Military Member, Veteran, or First Responder; SD = Standard deviation. Individual and household income data was missing for one intimate partner. Thus, the denominator for those variables was nine instead of 10 for the ITT sample, and six instead of seven for the completer sample. Additionally, category percentages for the following items were not mutually exclusive (i.e., some participants endorsed more than one category): Current MMVFR Status, Current Treatment.

### 2.2 Measures

Participants completed measures of demographics and mental healthcare utilization at baseline. Exclusion criteria were assessed using one item inquiring about past week suicidal thoughts (endorsement of such thoughts for moderate or long periods of time are exclusions) and one item inquiring about past suicide attempts and their dates (with a past year attempt as an exclusion). Intimate partner violence was assessed via three items derived from the Abuse Assessment Screen (Weiss et al., 2003) and the Partner Violence Screen (Feldhaus et al., 1997) wherein being hit, kicked, punched, hurt, or experiencing forced sexual activities by a partner in the past year, or not feeling physically safe in the relationship, were exclusion criteria.

#### 2.2.1 Primary outcomes

The Posttraumatic Stress Disorder Checklist-5 (PCL-5; Weathers et al., 2013) is a reliable and valid 20-item self-report measure of PTSD symptoms consistent with DSM-5 criteria (APA, 2013). The PCL exhibits high test-retest reliability and convergent validity with measures of related phenomena such as depression, anxiety, and functional impairment (Bovin et al., 2016). It was administered to assess for study inclusion criteria (i.e., elevated PTSD symptoms) and as the primary outcome measure (Cronbach α = 0.87). Participants with probable PTSD (i.e., PTSD+ participants) indicate the extent to which they have been bothered by various symptoms in the past month (for screening) or past week (for outcome measurement). At screening, a version of the PCL-5 that inquires about the nature of the traumatic event itself was included. Research team members reviewed participants' responses to these queries to determine whether the traumatic events that they reported were consistent with a DSM-5 Criterion A traumatic event (APA, 2013) as part of the eligibility screening process. This version of the PCL then asks participants to specifically base their responses regarding PTSD symptom queries on problems that started or got worse after their identified event. Partners also provide a collateral-report version of the PCL-5 which has been used in prior dyadic work (Cronbach α = 0.96) (e.g., Ennis et al., 2021).

Relationship satisfaction was assessed using the 4-item version of the Couples Satisfaction Index (CSI-4; Funk and Rogge, 2007) (PTSD+ participant α = 0.82; Partner α = 0.96). Participants are asked to rate the degree of happiness, perceived warmth, reward, and satisfaction in their relationship. The CSI-4 has strong convergent validity with other gold standard relationship satisfaction measures (Funk and Rogge, 2007).

#### 2.2.2 Secondary outcomes

PTSD+ participants and partners both completed the following secondary outcome measures: The *Client Satisfaction Questionnaire* (CSQ-8; Attkisson and Zwick, 1982) was used to measure overall satisfaction with the intervention (α = 0.96). The CSQ-8 is a reliable and valid tool for the evaluation of web-based interventions (Boß et al., 2016). The Client Satisfaction Questionnaire (CSQ-8) has demonstrated excellent test-retest reliability and construct validity, with less satisfied clients dropping out of programs earlier than satisfied clients (Larsen et al., 1979).

Partners also completed the *Significant Others Responses to Trauma Scale* (SORTS; Fredman et al., 2014), which is a 20-item scale that assesses partners' behavioral accommodation of PTSD symptoms (α = 0.84). The SORTS demonstrates strong internal consistency, high test-retest reliability, and associations with individual and relationship distress (Fredman et al., 2021). For example, it has a large and significant positive association between partners' perceptions of PTSD symptom severity as well as PTSD+ participants' depressive symptoms (Fredman et al., 2021).

*The Ineffective Arguing Scale* (Kurdek, 1994) was used to measure argumentativeness and conflict (α = 0.85). The Ineffective Arguing Inventory exhibits strong convergent validity with measures of relationship satisfaction and dissolution (Kurdek, 1994), and scores are strongly correlated in expected directions within couples (Kurdek, 1994).

*The Patient Health Questionnaire-9* (PHQ-9; Kroenke et al., 2001) was used to measure depressive symptoms (α = 0.85). The PHQ-9 has excellent test-retest reliability (*r* = 0.84) (Kroenke et al., 2001) and superior criterion validity compared to two other established depression screening questionnaires (Löwe et al., 2004a). It is also sensitive to change over time (Löwe et al., 2004b).

*The Generalized Anxiety Disorder-7* (GAD-7; Spitzer et al., 2006) was used to measure symptoms of generalized anxiety (α = 0.90). The GAD-7 has strong psychometric characteristics, including good test-retest reliability (Löwe et al., 2008; Spitzer et al., 2006) and convergent validity with other measures of anxiety and related constructs for worry and stress (Kertz et al., 2013; Rutter and Brown, 2017).

*The State-Trait Anger Expression Inventory-2 (STAXI-2) - Trait Anger Subscale (T-Ang)* (Spielberger, 1999) was used to measure anger (α = 0.80). The STAXI-2 is a reliable and valid tool with, for example, high concurrent validity with related measures of anger and aggression (Lievaart et al., 2016).

*The Trauma-Related Guilt Inventory* (TRGI; Kubany et al., 1996) was used to measure trauma-related guilt (PTSD+ participants only; α = 0.97). The TRGI has strong psychometric properties such as high test-retest reliability and convergent validity with measures of PTSD, depression, trait shame, social anxiety, and avoidance (Kubany et al., 1996).

*The Addiction Severity Index* (ASI; McLellan et al., 1980) was used to measure alcohol (α = 0.78) and drug (alpha not applicable) use. This measure inquires about the number of days alcohol was used, used to intoxication, and alcohol problems were experienced in the past seven days. It also inquires about the amount of money spent on alcohol and the extent to which alcohol problems have been bothersome in the past seven days, and the importance of treatment for these problems. The same questions are asked about with respect to drug use. Items are compiled into a composite measure of alcohol and drug use severity, respectively. However, only one participant had a non-zero composite score at each time point, so change in drug use could not be tested in this sample. Research suggests that the alcohol use subscale of the ASI correlates well and in expected directions with related measures (Appleby et al., 1997).

Finally, three items from the World Health Organization were selected based on their face validity and in alignment with other trials of online couple programs (Doss et al., 2016). These items measured 1) how satisfied participants were with their physical health; 2) their ability to function at work and complete household tasks; and 3) their overall quality of life (WHOQOL Group, 1998).

### 2.3 Intervention

Couple HOPES consists of seven interactive modules (see Table 2 for a brief overview of Couple HOPES content by module). Unlike CBCT for PTSD, Couple HOPES does not formally target PTSD-related beliefs in light of data suggesting that PTSD improves in CBCT for PTSD prior to the introduction of this content (Fredman et al., 2019; Monson et al., 2012) (see Monson et al., in press, for full intervention description including changes from CBCT for PTSD). Before each module, both partners complete self- or collateral-report measures of PTSD symptoms (PCL-5) and relationship satisfaction (CSI-4). Change is depicted via an automated graph that participants view when they log into their Couple HOPES portal. Each module consists of streamed video, interactive within-module exercises, and out-of-module practice assignments. Each partner has their own account for entering practice assignment responses that are connected to their partner's account. Five of ten couples also had access to the Couple HOPES mobile app to enter practice assignment responses, receive notifications, and use secure messaging with their partner and coach.

**Table 2:** Overview of couple HOPES module content.

Module	Module content
1	Psychoeducation on PTSD symptoms and relationship functioning
2	Safety building in relationships and introducing skills to manage relationship conflict
3	Communication skills, psychoeducation regarding avoidance in PTSD
4	Approaching situations, conversations, and experiences that are avoided as a result of PTSD symptoms
5	Sharing feelings
6	Sharing thoughts
7	Consolidating intervention gains and relapse prevention

*Note.* PTSD = Posttraumatic stress disorder.

Couples are assigned a coach who is trained to adhere to the coaching manual to ensure fidelity to the intervention. Coaches are also trained in crisis management strategies and supervised by the study investigators. The coaching manual was written with the intention for coaching to be delivered by paraprofessionals, and coaches in this study were study investigators, graduate students, and bachelors-level team members. Coaches attended weekly coaching meetings run by study investigators wherein recordings of coaching calls and the progress of couples were reviewed. Coaches are provided with feedback in these meetings to enhance adherence to the manual. All coaches attended these weekly meetings, reviewed the coaching manual, and submitted a mock call recording with a simulated couple for review to the investigators prior to being designated as a study coach. In addition, coaches were orally tested regarding crisis management strategies prior to being designated as a study coach.

Couples meet with coaches for four scheduled calls that occur via secure videoconferencing after modules one, three, five, and seven. The main goals of the calls are to motivate the couple for intervention engagement and adherence, troubleshoot skill implementation or technical issues, and reinforce the couple for their successes. The first scheduled call lasts up to 20 min, and subsequent calls last up to 15 min. Couples also have the option of receiving one additional as-needed call at any time during the program. Scheduled calls occur either weekly or bi-weekly, depending on participants' preference for pacing the program. As-needed calls are implemented to troubleshoot or discuss a range of potential issues such as a couple considering dropping out of the program, a lack of improvement in PTSD scores, a lack of practice assignment or module completion, or technical issues with the platform. The content of these calls thus varies from addressing couple's concerns about the intervention, troubleshooting non-adherence, clarifying program content, or providing instructions in the use of the platform. Coaches are also accessible via secure messaging during the intervention.

### 2.4 Procedures

All study procedures were approved by relevant institutional review boards. Participants were recruited from social media advertisements and community outreach. Interested participants signed up for the study through the Couple HOPES website and were sent separate screening surveys to determine eligibility. Eligible couples received an online consent form and were asked to complete an online baseline assessment. Once enrolled, couples were contacted by their assigned coach to schedule their first coaching call. In addition to completion of the PCL-5 and CSI-4 prior to each module, participants completed all outcome measures at baseline, mid-intervention (i.e., after the completion of Module three and its associated coaching call), and post-intervention (i.e., after the completion of Module seven and its associated coaching call, or after eight weeks since enrollment, whichever came first).

Participants were considered “non-completers” if they did not complete all seven modules within eight weeks. At this point, they no longer had access to coaching but retained platform access (and the ability to progress through it) for 12 months following their date of withdrawal. If couples did not complete any modules throughout the 8-week study period, or withdrew from the intervention, then the mid-assessment was provided at the end of the study period (i.e., 8-weeks after enrollment), and the post-assessment was provided one week after the mid-assessment. Participants were compensated for these assessments in the form of gift cards.

### 2.5 Data analytic plan

Feasibility and acceptability of the program were assessed via the number of couples that completed the program and self-reported satisfaction with the program post-intervention. Program efficacy was tested via change in primary and secondary outcomes.^1^ Efficacy was examined using both statistical change (pre-post *t*-test) and clinically significant change criteria. Consistent with recommendations (Cumming, 2013; Feingold, 2009), effect size estimates were computed by dividing the mean pre-post change by the standard deviation of the pre-intervention score. A Hedge's *g* correction was applied due to standardized effect size estimates being inflated at small samples (Hedges, 1981). Efficacy was examined for all outcomes (other than program satisfaction) completed by PTSD+ participants and collateral-reports of PTSD symptoms, relationship satisfaction, and accommodation of PTSD symptom measures completed by partners. Of the six non-completer individuals (three couples), three did not complete any assessments after the initial assessment, and the other three completed only two of three assessments (one post-, two mid-intervention). Due to the missing data from non-completers and the low sample size prohibition on methods that can handle partially-missing data (e.g., multilevel models), we focused on completer analyses to assess preliminary evidence of program efficacy.^2^

Clinically significant change was assessed for primary outcomes using the formulae and categories from Jacobson and Truax (1991): recovered (reliably improved and crossed diagnostic threshold), improved (reliably improved but did not cross diagnostic threshold), worsened (reliably deteriorated), or unchanged (no reliable change). Reliable change was determined using Jacobson & Truax's formula, in which r_xx_ is from internal reliability (consistent with more recent recommendations; Lambert and Ogles, 2009), and *SD* is the pre-intervention standard deviation of the full sample (PTSD+ participant = 11.8; Partner collateral-reported PTSD symptoms = 15.1; relationship satisfaction = 3.37). Diagnostic threshold and internal reliability were based on measure validation studies and were, respectively, 33 and 0.91 for PTSD symptoms (Wortmann et al., 2016), and 13.5 and 0.94 for relationship satisfaction (Funk and Rogge, 2007). In absence of a large-sample estimate for internal reliability of partner collateral-reported PTSD symptoms, we used the same threshold as for the PTSD+ participant version, which was slightly more conservative than that (8.37) obtained using the current sample's internal reliability (0.96). Therefore, the threshold of reliable change was 9.8 for PTSD symptoms and 2.3 for relationship satisfaction.

## 3 Results

Of the ten enrolled couples, seven completed the program, and mean satisfaction with the program for completers was 3.4 out of 4 (*SD* = 0.7) for PTSD+ participants and 3.7 out of 4 (*SD* = 0.4) for their intimate partners. Of the three couples who did not complete the program, two never began the program or responded to the study team's communication attempts. The other couple withdrew after a medical event that was not study-related and did not progress past the third module. Table 3 includes means and standard deviations for study variables at each time point, as well as statistical results and effect size estimates for pre-post change for completers. There were significant, large effect size improvements in the PTSD+ participants' self-reported PTSD symptoms (*g =* 0.80) and perceived health (*g* = 1.13) and marginally significant, medium effect size improvements in quality of life (*g* = 0.62). Partners' relationship satisfaction also significantly improved with a medium effect size *(g* = 0.68). There were medium effect size improvements in partners' accommodation of PTSD symptoms *(g* = 0.56) and PTSD+ participant's depression *(g* = 0.53), small to medium effect size improvements in argumentativeness *(g* = 0.43), and small effect size improvements in anger *(g* = 0.31), generalized anxiety (*g* = 0.31), and work functioning (*g* = 0.25); however, these effects were not statistically significant in the small sample. Finally, collateral-reported PTSD symptoms, and PTSD+ participants' relationship satisfaction, trauma-related guilt, and alcohol use showed negligible effect size changes (*g*s range from −0.07 to 0.12).

**Table 3:** Means and standard deviations for study variables at each time point, *t*-test results, and pre-post effect size estimates for completers.

Variable	Measure range	Pre *M* (*SD*)	Mid *M* (*SD*)	Post *M* (*SD*)	*t*_pre-post­_ (6) =	*p*	Within-group *Hedge's g*
Participant with probable PTSD							
PTSD symptoms	0–80	47.3 (11.9)	38.4 (7.6)	34.0 (9.9)	2.79	0.03	0.80
Relationship satisfaction	0–21	14.0 (3.5)	13.4 (4.1)	14.1 (3.6)	0.15	0.89	0.02
Argumentativeness	8–40	24.0 (7.6)	21.0 (2.9)	19.7 (4.4)	1.97	0.10	0.43
Depression	0–27	13.6 (3.8)	10.7 (4.3)	10.9 (4.8)	1.46	0.20	0.53
GAD	0–21	10.7 (2.2)	11.4 (4.6)	9.4 (4.0)	0.72	0.50	0.31
Anger	10–40	27.1 (6.5)	25.6 (4.6)	24.6 (4.9)	1.82	0.12	0.31
Trauma-Related Guilt	0–128	49.6 (34.0)	50.6 (34.4)	52.4 (37.5)	−0.45	0.67	−0.07
Alcohol use	0–1	0.23 (0.36)	0.26 (0.27)	0.22 (0.29)	0.05	0.96	0.02
Perceived Health	1–5	1.9 (1.1)	2.3 (1.3)	3.3 (1.0)	3.33	0.02	1.13
Work Functioning	1–5	2.6 (1.3)	2.6 (1.0)	3.0 (1.4)	0.75	0.48	0.25
Quality of Life	1–5	2.6 (1.0)	3.1 (0.7)	3.4 (1.1)	2.12	0.08	0.62

Partners							
PTSD symptoms (Partner collateral-report)	0–80	29.1 (8.5)	30.0 (8.8)	26.6 (15.8)	0.34	0.75	0.12
Relationship satisfaction	0–21	11.3 (3.1)	12.0 (2.7)	14.0 (3.8)	2.96	0.03	0.68
Accommodation	0–76	15.1 (9.5)	10.5 (7.6)	8.9 (6.1)	1.33	0.23	0.56

*Note*. *Hedges g* is coded so that positive values represent improvement. PTSD = Posttraumatic stress disorder; GAD = generalized anxiety symptoms.

Regarding clinically significant change in the seven intervention completers, two PTSD+ participants' self-reported PTSD symptoms were categorized as recovered and three reliably improved. Partners' collateral-report ratings of PTSD symptoms were categorized as recovered in one case, improved in two cases, and deteriorated in one case. Finally, changes in the PTSD+ participants' relationship satisfaction were considered recovered in one case and deteriorated in one case, and the partners' relationship satisfaction was recovered in three cases and improved in one case.

## 4 Discussion

This study is the first test of the feasibility, acceptability, and efficacy of Couple HOPES in 10 couples wherein one member was a MMVFR with probable PTSD. Low dropout rates on par with CBCT for PTSD (Liebman et al., 2020), high levels of user satisfaction, and a series of efficacy tests generally supported the feasibility, acceptability, and efficacy of the program. Specifically, PTSD+ participants exhibited large effect size improvements in PTSD and perceived health, and medium-sized improvements in quality of life, argumentativeness, and depression. Moreover, intimate partners reported medium effect size improvements in relationship satisfaction and accommodation.

The improvements in PTSD symptoms found in this initial study are large. However, this is a small sample of “early intervention adopters.” Moreover, although it is encouraging that PTSD+ participants reported substantial reductions in PTSD symptoms, it is unclear why collateral-reported PTSD symptoms were not comparably improved. A central focus of Couple HOPES and CBCT for PTSD involves providing psychoeducation regarding the nature of PTSD and its impact on relationships, and facilitation of communication about these issues between partners. Our anecdotal observation of these cases suggests that some partners' collateral-reports of PTSD *increased* over the course of the program as a result of enhanced sensitization to the disorder and its sequelae rather than worsening of PTSD per se. Indeed, the increase in relationship satisfaction reported by intimate partners suggests that such heightened awareness and communication may enhance well-being, even if it does not appear to them to improve PTSD.

PTSD+ participants' relationship satisfaction did not improve over the course of the program, while partners' did. However, on average, PTSD+ participants did not report relationship satisfaction in the distressed range at baseline, although their intimate partners did, leaving less room for improvement. These findings suggest that, while PTSD+ participants may primarily benefit from Couple HOPES by improving PTSD symptoms, their partners may primarily benefit from enhancing their relationship satisfaction. This finding is consistent with research on CBCT for PTSD that shows that partners may have most robust improvements in their relationship satisfaction from the intervention (Liebman et al., 2020). More research is needed to ascertain whether PTSD+ participants with lower relationship satisfaction would experience increases in it over the course of the program.

Although these preliminary findings are encouraging, they must be contextualized within the study's limitations. Most notably, these results are derived from a case series with a small sample size. Thus, findings are nascent and must be interpreted with caution. This is particularly true for statistical significance findings, wherein statistically significant effects or lack thereof are vulnerable to issues of power and outliers. Future work testing the efficacy of Couple HOPES in a larger sample and compared to inactive and active controls is needed to gain a clearer understanding of its efficacy.

Moreover, because this data is from the first cases to receive Couple HOPES, coaching and study protocols were refined throughout it. For example, a scheduled fourth coaching call was added early on in the intervention in lieu of a second extra as-needed call; the length of the first coaching call was increased from 15 min to 20; coaching content was refined to focus less on symptom and relationship satisfaction improvement and intervention content and more on program adherence and engagement; and the mobile app was not available for all couples. These changes strengthened the program but also somewhat compromised study internal validity. Future testing with a fixed coaching manual and platform is needed. Finally, primary study analyses focused on completers rather than the ITT sample given that identifying the effect of an intervention for those who receive it is of primary importance for a newly developed program. We also did not conduct follow-up assessments, and thus the maintenance of gains from Couple HOPES remains unclear. Subsequent testing using an ITT sample with a follow-up period is a critical next step for this work.

### 4.1 Conclusions

Despite these limitations, this study suggests that Couple HOPES may be an acceptable, feasible, and efficacious means of reducing PTSD symptoms and enhancing relationship satisfaction in MMVFR and their partners. These promising findings are welcomed in the current mental health landscape in which evidence-based mental health services are difficult to access for many and increasingly demanded. Our preliminary findings suggest that Couple HOPES may be one method to realize the promise of access evidence-based PTSD interventions in a time when they are most needed.

## Role of the funding source

The initial development and testing of Couple HOPES have been funded by the Government of Canada's Department of National Defence [IDEaS_CP-0971]. Couple HOPES testing was also funded by the Canadian Institute for Military and Veterans Health Research. Other than approving it for funding, these organizations were not involved in the study design, data collection, data analysis, or interpretation of data.

## Declaration of competing interest

The authors declare the following financial interests/personal relationships which may be considered as potential competing interests: Dr. Candice Monson receives royalties from Guilford Press related to the publication of the treatment manual from which Couple HOPES was adapted.

## Footnotes

1 The clinical trial registration (Clinicaltrials.gov identifier NCT04231578) also included change in drug use as an additional outcome. However, only one participant had a non-zero composite score at each time point, so change in drug use could not be tested in this sample.

2 As a sensitivity analysis, we also approximated intention-to-treat analyses (ITT) in the following way. Two noncompleters completed the pre- and mid-intervention assessments, and their post assessment was imputed by using the pre- to mid-intervention trendline for each outcome to estimate the outcome at post-intervention. These two individuals, plus a third noncompleter whose post data was not missing, were then combined with the rest of the sample for the ITT analyses, which were again conducted with a dependent-groups *t*-test. This approach is conceptually similar to how a multilevel model incorporates cases with partially missing data, but without the shrinkage component. ITT results were consistent with completer analyses and did not meaningfully alter study conclusions.
